# Supplementary material for: Photo-Isomerization Kinetics of Azobenzene Containing Surfactant Conjugated with Polyelectrolyte
Source: Molecules. 2020 Dec 22;26(1):19. doi: 10.3390/molecules26010019 (PMC7793112; doi:10.3390/molecules26010019)
Supplement: Supplementary file 1 [file molecules-26-00019-s001.pdf]

Supplementary Material

Photo-Isomerization Kinetic of Azobenzene Containing Surfactant Conjugated with Polyelectrolyte

Anjali Sharma, Marek Bekir, Nino Lomadze and Svetlana Santer*

Institute of Physics and Astronomy, University of Potsdam, 14476 Potsdam, Germany

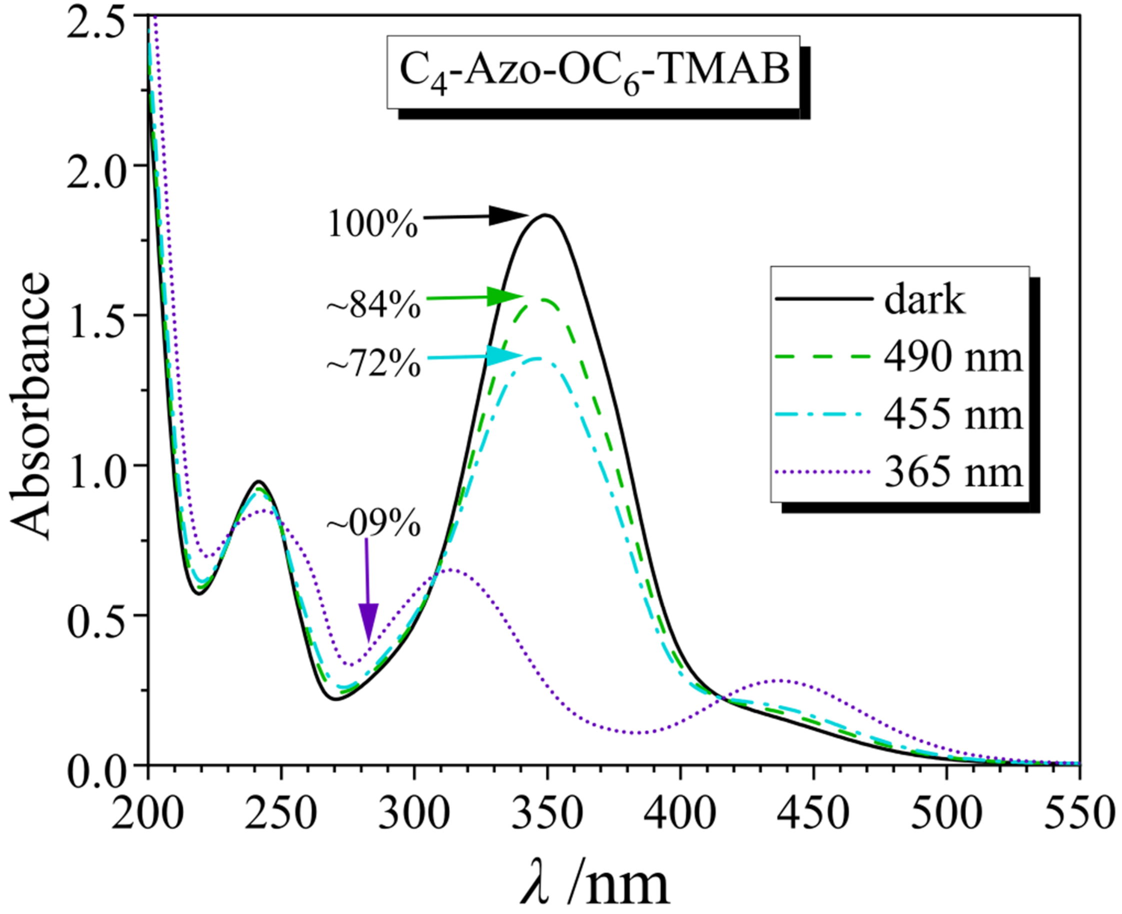

Figure S1. UV-VIS absorption spectra of the surfactant in the dark and under irradiation with green, blue and UV light (λ = 490nm, λ = 455nm, λ = 365nm) for 0.1mM surfactant concentration. The percentage of *trans* isomers present in the system is depicted next to each curve.

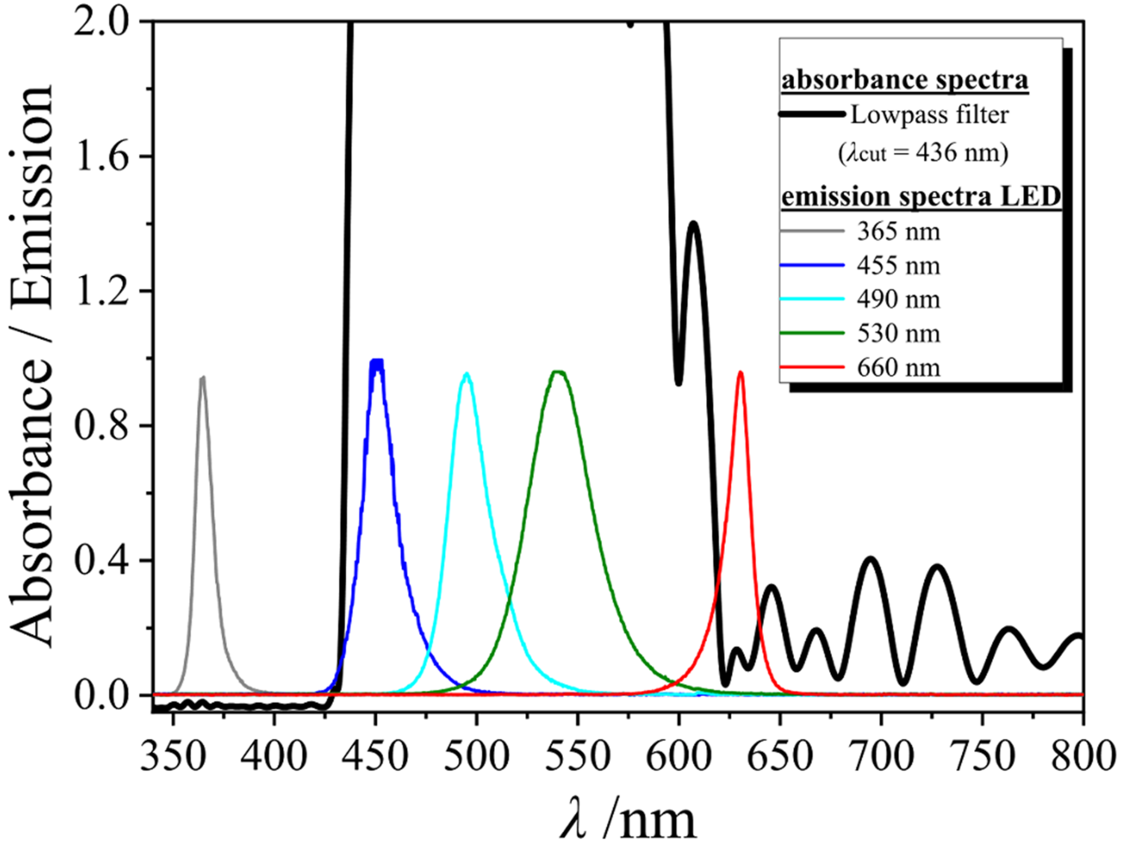

Figure S2. Transmission spectrum of the used low-pass filter and the emission spectra of the used LEDs: (UV, λ_{UV} = 365 nm), (blue, λ_B = 455 nm), (turquoise, λ_T = 490 nm), (green, λ_G = 530 nm).

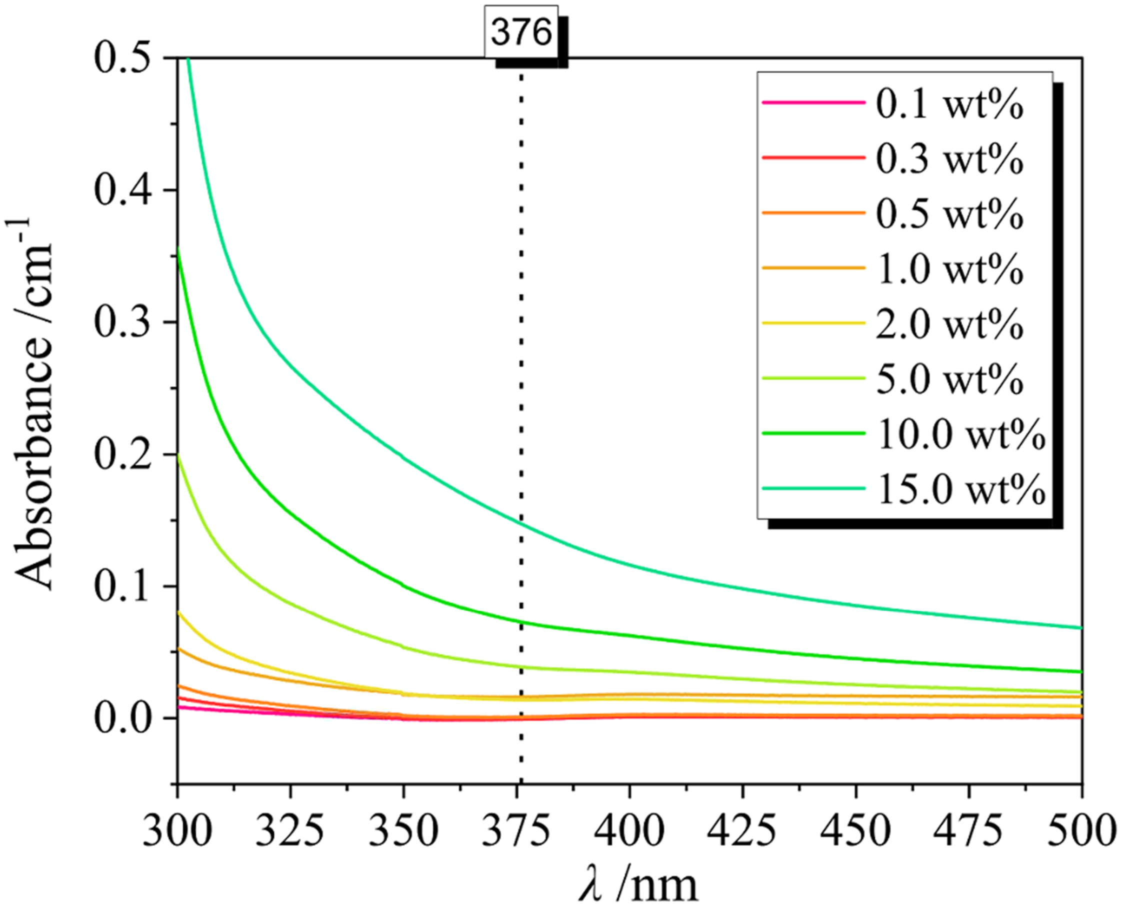

Figure S3. Absorption spectra of pure PAA for different concentration to read out the value of Abss.

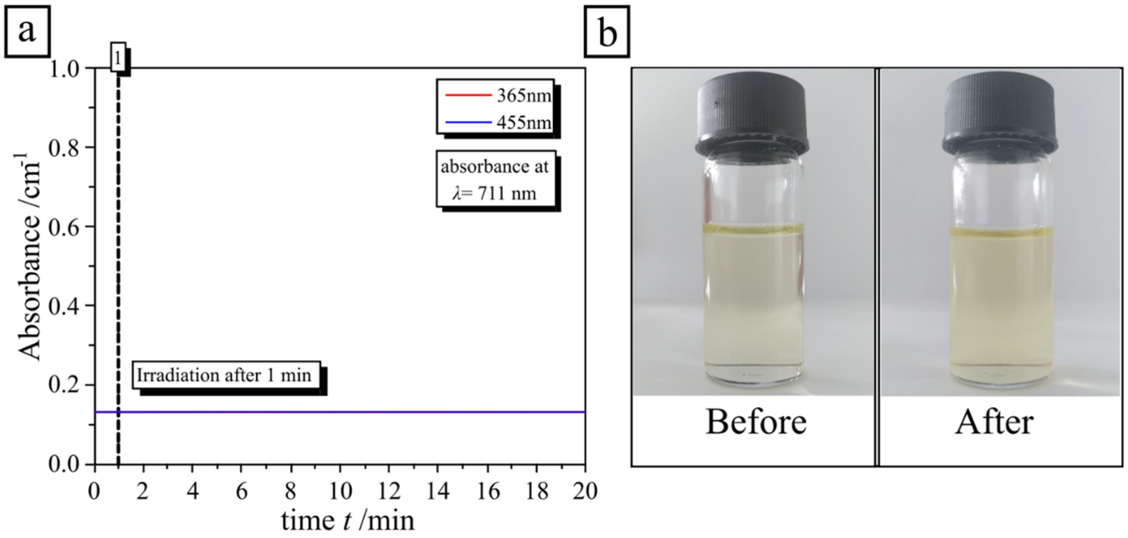

Figure S4. (a) Absorbance acquired at 711nm of PAA-AzoC$_6$ complex as a function of irradiation time for two different wavelengths, UV and blue (c_{PAA} = 15wt%, c_{AzoC6} = 0.1 mM, I = 1mW/cm^2). (b) Photo of the sample before and after irradiation. (λ_{UV} = 365 nm).

Section 1. Calculation of the Z ratio between PAA and surfactant

$$Z = \frac{N_+}{N_-} = \frac{c_+}{c_-} \qquad \text{(S1)}$$

$$c_+ = c_{AzoC6} = 0.1 \text{ mM} \qquad \text{(S2)}$$

$$c_- = \alpha \cdot c_{PAA} \qquad \text{(S3)}$$

$$c_{PAA} = \frac{m_{PAA}}{MW_{PAA}(monomer) \cdot V_S} \qquad \text{(S4)}$$

$$MW_{PAA}(monomer) = 0.5 \cdot MW_{PAA}(OH) + 0.5 \cdot MW_{PAA}(ONa) = 113.12 \text{ g/mol} \qquad \text{(S5)}$$

$$m_{\text{PAA}} = \frac{\frac{wt\%_{PAA}}{100}}{1-\frac{wt\%_{PAA}}{100}} \cdot \frac{\rho_{sample}}{V_S} \text{ with } \rho_{sample} \sim 1\frac{\text{g}}{\text{mL}} \qquad \text{(S6)}$$

Table S1. Concentration of PAA monomers (c_{PAA}) in units of wt% and mM, concentration of anionic charges (c_-) in units of mM, Z ratio (Z).

c_{PAA}	c_{PAA}	c_-	Z
wt%	mM	mM	
0	0	0	—
0.1	9	4	$2.273 * 10^{-2}$
0.3	27	13	$7.519 * 10^{-3}$
0.5	44	22	$4.505 * 10^{-3}$
1	89	45	$2.242 * 10^{-3}$
2	180	90	$1.109 * 10^{-3}$
5	465	233	$4.299 * 10^{-4}$
10	982	491	$2.036 * 10^{-4}$
15	1560	780	$1.282 * 10^{-4}$
20	2210	1105	$9.050 * 10^{-5}$

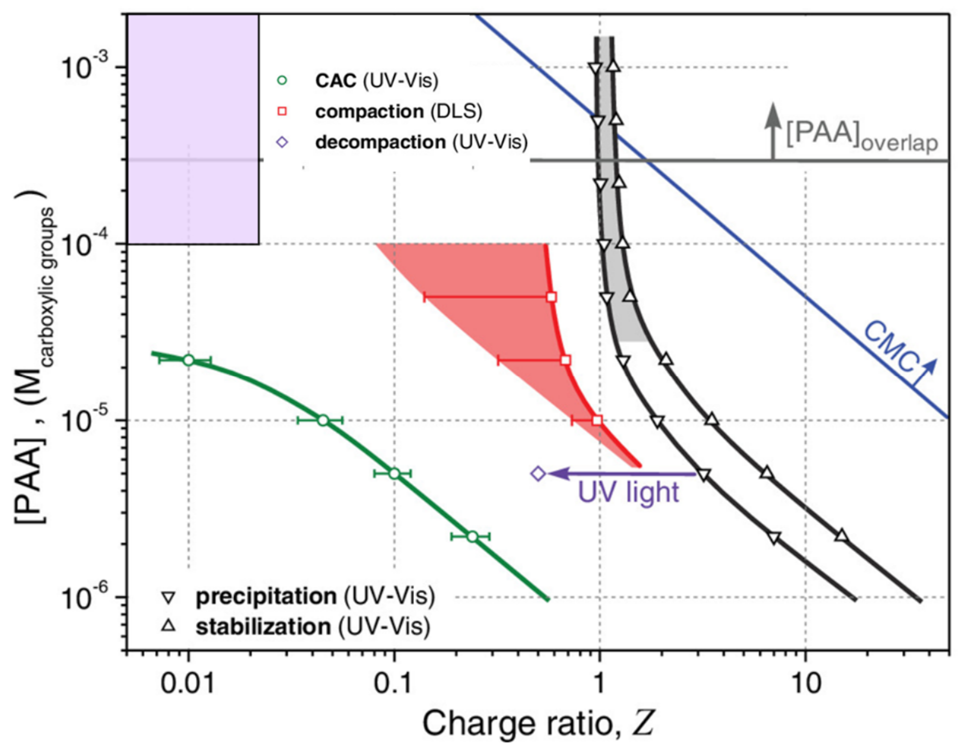

Figure S5. Phase diagram for PAA–C_4-Azo-OC_6TMAB complexes. Charge ratio Z is the molar ratio of concentrations of C_4-Azo-OC_6TMAB and PAA carboxylic groups as reported in Ref. 32 (main text). The highlighted region in purple illustrates the concentration range used in this study.

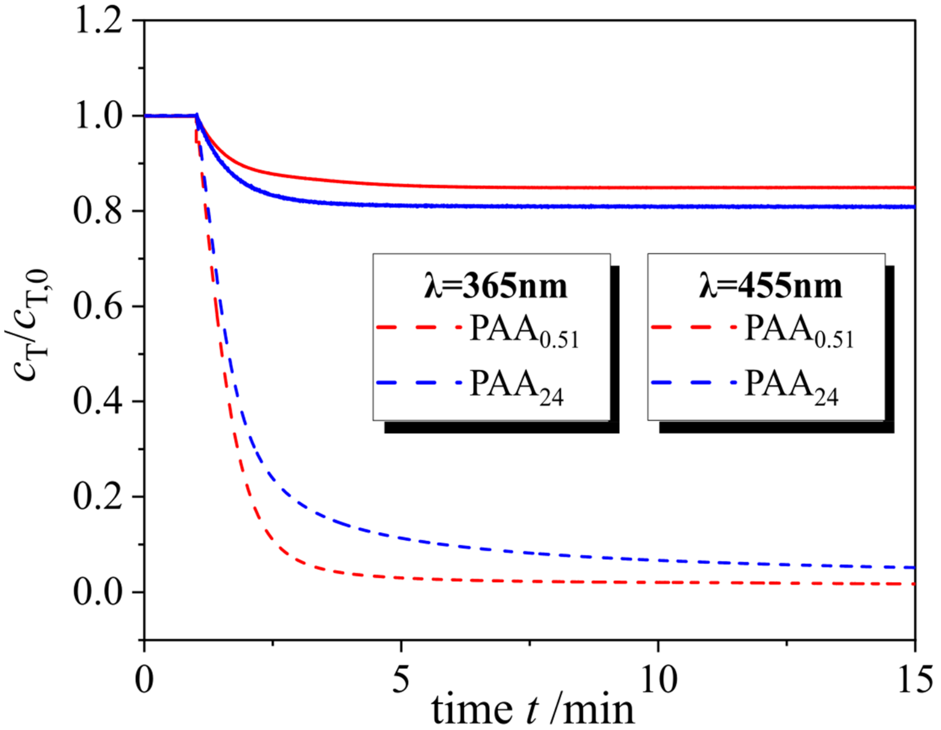

Figure S6. Dependence of the *trans*-isomer concentration, $c_T/c_{T,0}$, in the polymer-surfactant complexes on irradiation time for two wavelength UV (dashed lines) and blue irradiation (solid lines). Rate constants for forward and reverse reaction under UV and blue irradiation are tabulated below.

Table S2. Molecular weight of PAA $M_{W,PAA}$, rate constant of trans-cis and vica versa k_{TC} and k_{CT}.

$M_{W,PAA}$ g/mol (·10^{-3})	k_{TC} $cm^2(mW·s)^{-1}$	k_{TC} error	k_{CT} $cm^2(mW·s)^{-1}$	k_{CT} error
PAA $_{5.1}$	0.00308	2.92 · 10^{-04}	0.01775	0.00203
PAA $_{24}$	0.00532	4.87 · 10^{-04}	0.02246	0.00246

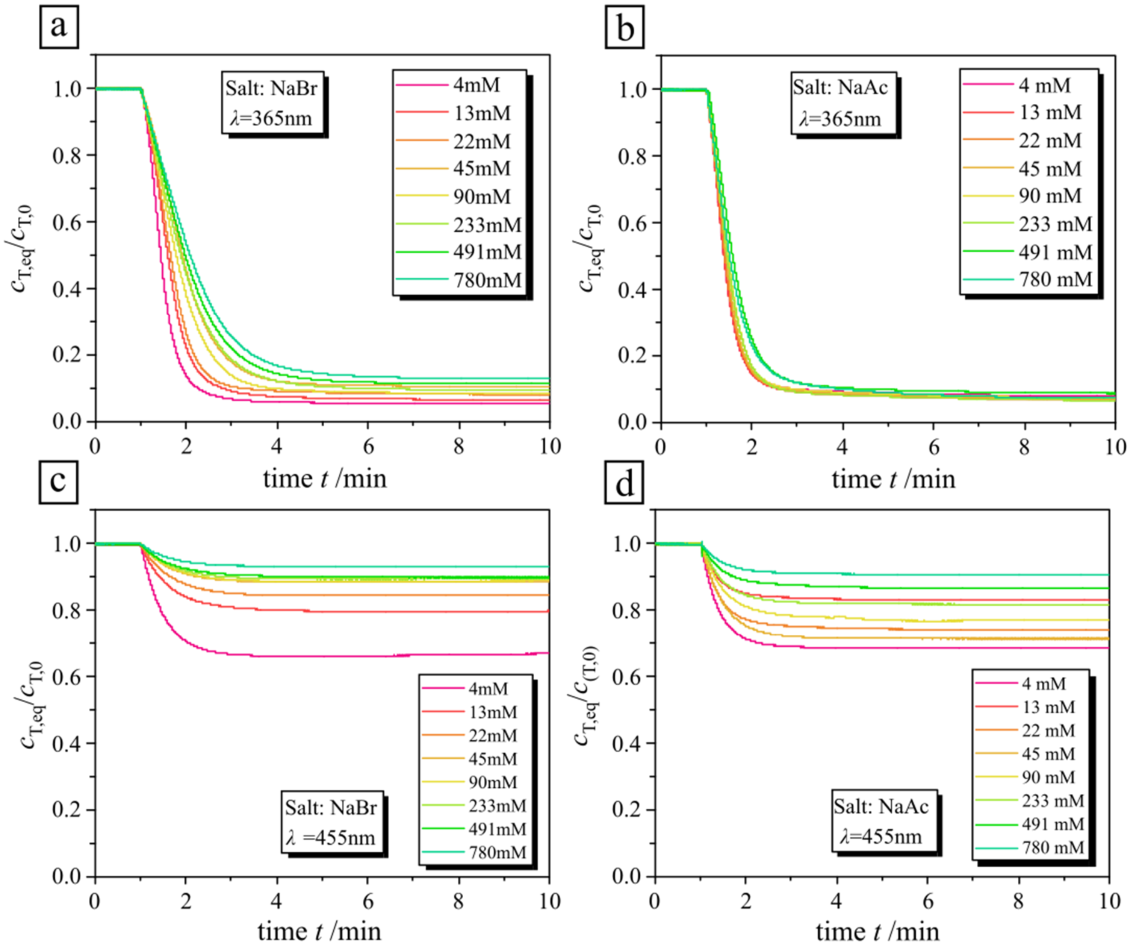

Figure S7. Time resolved $c_{T,eq}/c_{T,0}$ of salt-AzoC$_6$ mixture as a function of time for concentrations varying from 4mM to 780mM, monitored at 376nm. (a) Absorption of NaBr-surfactant during irradiation with UV. (b) Absorption of NaAc-surfactant during irradiation with UV. (c) Absorption of NaBr-surfactant during irradiation with blue LED. (d) Absorption of NaAc-surfactant during irradiation with blue LED.

Table S3. Tabulated data for the rate constants for all salts as a function of salt concentration.

	λ=365nm						λ=455nm					
	NaCl*		NaBr		NaAc		NaCl*		NaBr		NaAc	
**Salt conc**	k_{TC}	k_{CT}	k_{TC}	k_{CT}	k_{TC}	k_{CT}	k_{TC}	k_{CT}	k_{TC}	k_{CT}	k_{TC}	k_{CT}
**mM**	$cm^{-2}(mW\cdot s)^{-1}$											
4	0.03048	0.00286	0.04261	0.00244	0.0438	0.00368	0.01101	0.02232	0.00914	0.01864	0.0131	0.02857
13	0.02523	0.00255	0.03382	0.00241	0.0443	0.00302	0.00559	0.02219	0.00999	0.02334	0.00657	0.03219
22	0.02537	0.00241	0.03398	0.00305	0.0442	0.00328	0.00426	0.02352	0.01079	0.02254	0.00857	0.02476
45	0.02152	0.00229	0.02127	0.00254	0.0456	0.00317	0.0027	0.02111	0.01031	0.02303	0.00956	0.02378
90	0.02024	0.00199	0.02384	0.0022	0.043	0.00333	0.00246	0.01976	0.00997	0.02336	0.00732	0.02473
233	0.01984	0.00239	0.01797	0.00187	0.0422	0.00287	0.00232	0.0199	0.00371	0.0201	0.00573	0.02571
491	0.02007	0.00215	0.01717	0.00221	0.0338	0.00323	0.00223	0.01999	0.004	0.01981	0.00459	0.02319
780	0.02568	0.0021	0.01612	0.0024	0.0368	0.00291	0.00187	0.02591	0.00373	0.0171	0.00265	0.02513

Table S4. Tabulated data for R^2, Reduced Chi-Sqr, τ and τ_{error} for PAA and NaBr.

	PAA + Salt (NaBr)							
	**365nm**				455nm			
**Salt conc**	**Adj. R^2**	**Reduced Chi-Sqr**	**τ**	**τ_{error}**	Adj. R^2	Reduced Chi-Sqr	τ	τ_{error}
mM								
4	0.98871	6.42E-04	4.48E-01	1.36E-03	0.99841	6.21E-06	0.49275	5.89E-04
13	0.98686	6.44E-04	5.91E-01	1.95E-03	0.99676	5.00E-06	0.68847	0.00116
22	0.9875	6.30E-04	6.31E-01	2.17E-03	0.9922	7.21E-06	0.68701	0.0018
45	0.98897	9.33E-04	8.99E-01	2.78E-03	0.98114	9.35E-06	0.69192	0.00284
90	0.99245	3.04E-04	8.02E-01	2.03E-03	0.98052	9.27E-06	0.69696	0.00291
223	0.99561	2.06E-04	9.46E-01	1.88E-03	0.97918	8.76E-06	0.73747	0.00318
491	0.99513	2.21E-04	1.01E+00	2.13E-03	0.97975	7.56E-06	0.84077	0.00359
780	0.99497	2.13E-04	1.09E+00	2.36E-03	0.93833	9.97E-06	0.73997	0.00562

Table S5. Tabulated data for R^2, Reduced Chi-Sqr, τ and τ_{error} for PAA and NaAc.

PAA + Salt (NaAc)								
	**365nm**				455nm			
**Salt conc mM**	**Adj. R^2**	**Reduced Chi-Sqr**	**τ**	**τ_{error}**	Adj. R^2	Reduced Chi-Sqr	τ	τ_{error}
4.42451	0.97423	1.30E-03	4.31E-01	1.84E-03	0.99968	7.79E-07	0.4019	1.99E-04
13.30016	0.97967	9.51E-04	4.36E-01	1.65E-03	0.98691	5.98E-06	0.47078	0.00152
22.21148	0.97907	9.93E-04	4.49E-01	1.72E-03	0.99083	1.30E-05	0.46173	0.00124
44.64732	0.9761	1.21E-03	4.73E-01	1.93E-03	0.99965	8.67E-07	0.50159	2.63E-04
90.20581	0.97655	1.17E-03	5.04E-01	2.04E-03	0.99048	1.50E-05	0.61275	0.00169
232.63605	0.99208	2.40E-04	4.91E-01	1.15E-03	0.99746	1.49E-06	0.52667	7.45E-04
491.12054	0.98956	3.98E-04	5.99E-01	1.60E-03	0.98296	5.29E-06	0.64153	0.00238
780.01498	0.99495	1.13E-04	5.68E-01	1.05E-03	0.98998	1.45E-06	0.55542	1.59E-03

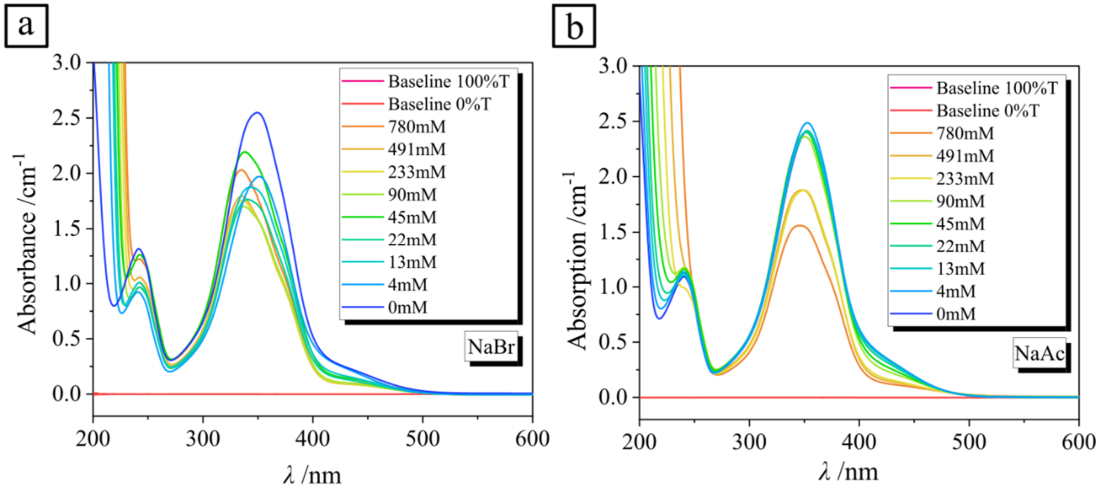

Figure S8. Absorption spectra of NaBr (a) and NaAc (b) for concentrations ranging between 0 to 780mM.

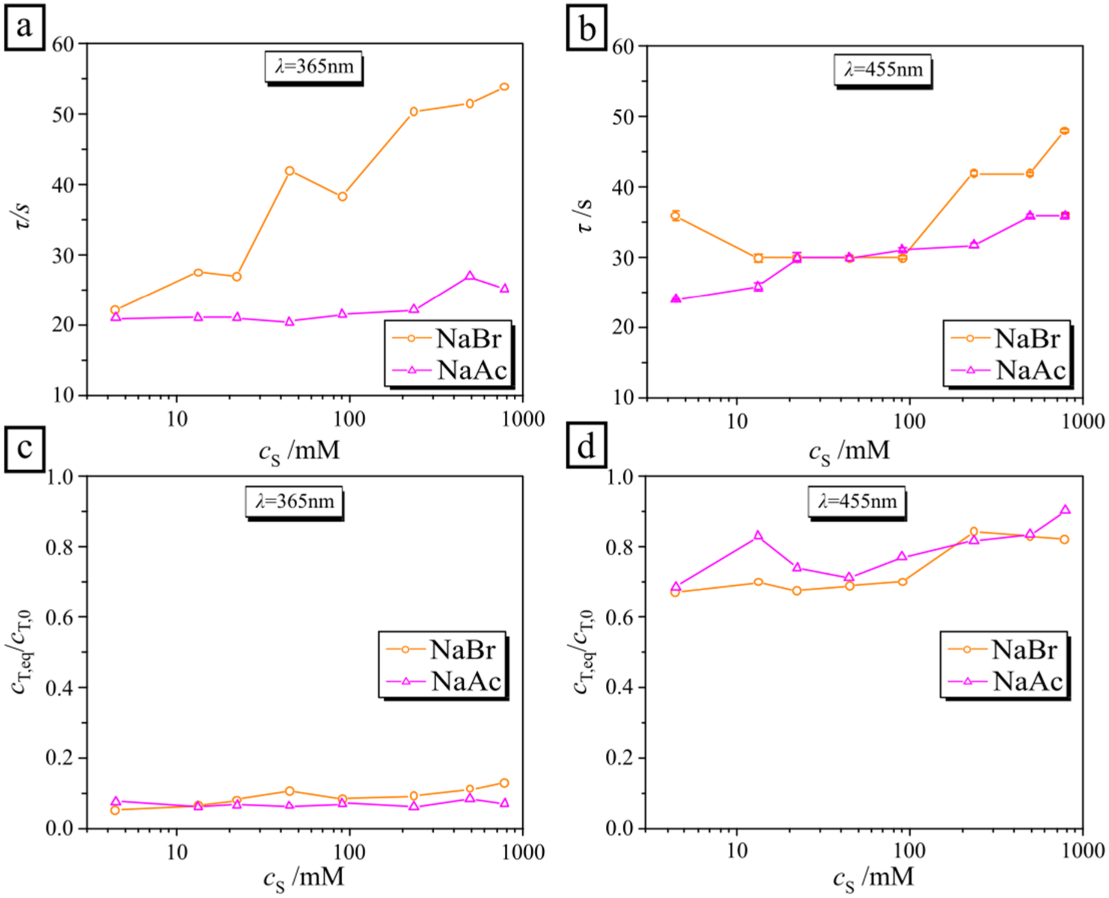

Figure S9. Kinetic measurements for salt-surfactant complex: (a) Time of decay for NaBr and NaAc during UV irradiation. (b) Time of decay for NaBr and NaAc during blue irridiation. (c) *Trans* ratio for NaBr and NaAc during UV irradiation. (b) *Trans* ratio for NaBr and NaAc during blue irradiation.

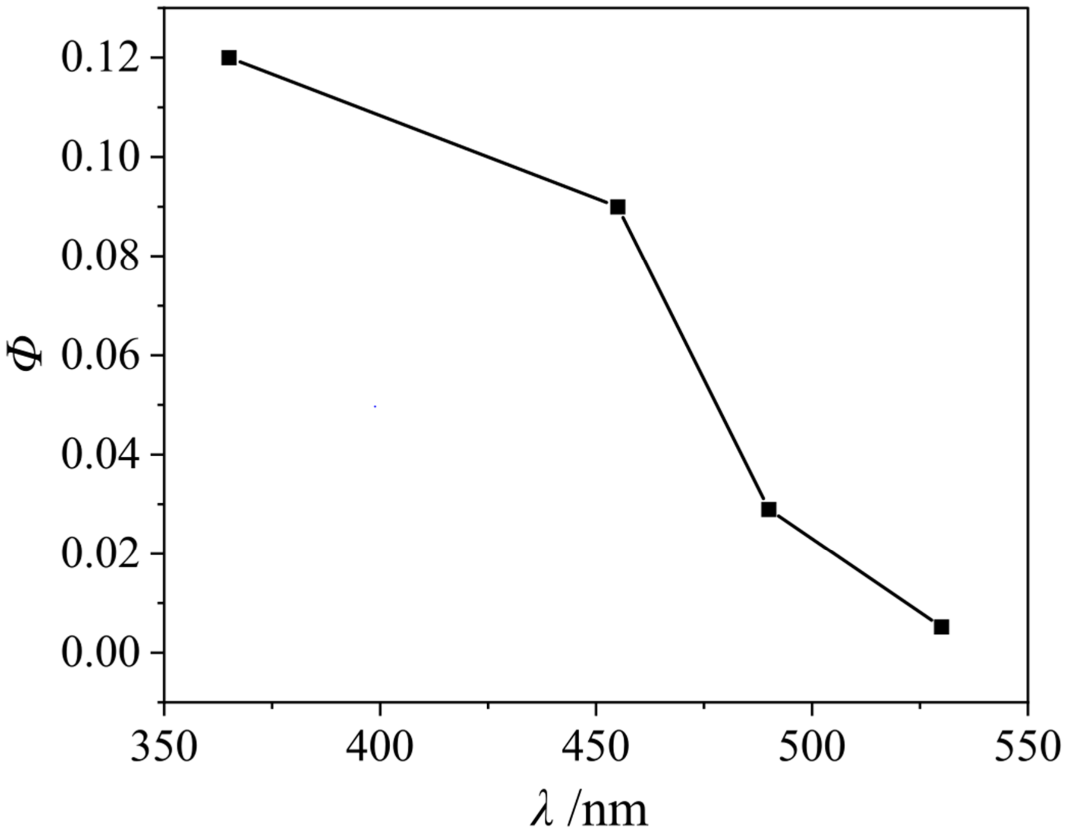

Figure S10. Quantum yields of isomerization at different wavelengths of irradiation.

Table S6. Tabulated data for the rate constants for different PAA contents under UV and Green irradiation.

PAA content	λ=365nm		λ=455nm	
	k_{TC}	k_{CT}	k_{TC}	k_{CT}
mol%	$cm^{-2}(mW{\cdot}s)^{-1}$			
0	0.03267	0.00205	0.008	0.01176
0.1	0.01595	0.00216	0.00251	0.01416
0.5	0.01738	0.00222	0.00382	0.01999
1	0.0183	0.00202	0.00294	0.01558
2	0.01886	0.00198	0.00262	0.01404
5	0.01998	0.00224	0.00289	0.01605
10	0.01839	0.00245	0.00272	0.0158
15	0.01658	0.00194	0.00268	0.01584

Table S7. Tabulated data for R^2, Reduced Chi-Sqr, τ and τ_{error} for different PAA contents under UV and Green irradiation.

PAA concentration dependence								
	365nm				455nm			
PAA content	Adj. R^2	Reduced Chi-Sqr	τ	τ_{error}	Adj. R^2	Reduced Chi-Sqr	τ	τ_{error}
wt%								
0	0.98321	0.00112	0.55009	0.00186	0.99909	3.95E-06	0.90793	5.85E-04
0.1	0.99382	1.64E-04	1.05E+00	2.26E-03	0.98536	6.93E-06	0.94074	0.00256
0.5	0.99528	1.73E-04	9.96E-01	1.84E-03	0.99643	2.37E-06	0.81222	0.00106
1	0.9937	1.59E-04	9.29E-01	2.01E-03	0.9998	1.27E-07	0.74972	2.28E-04
2	0.9958	9.35E-05	8.96E-01	1.56E-03	0.99164	5.11E-06	0.82284	0.00171
5	0.98426	7.83E-04	1.00E+00	3.21E-03	0.99556	2.59E-06	0.821	0.00121
10	0.98415	6.96E-04	1.07E+00	3.24E-03	0.99835	9.48E-07	0.85938	7.80E-04
15	0.98882	5.03E-04	1.14E+00	3.19E-03	0.99813	1.10E-06	0.8843	8.76E-04

Table S8. Tabulated data for the rate constants for different intensities under UV and Green irradiation.

**Intensity**	**λ=365nm**		**λ=455nm**	
	$\mathbf{k_{TC}}$	$\mathbf{k_{CT}}$	$\mathbf{k_{TC}}$	$\mathbf{k_{CT}}$
**mW·cm^{-2}**	**cm^{-2}(mW·s)$^{-1}$**			
0.5	0.02984	0.0035	0.0036	0.02021
1	0.02483	0.00295	0.00303	0.0178
2	0.0187	0.00213	0.0042	0.02268
4	0.01879	0.00205	0.00328	0.01756
8	0.01881	0.00203	0.00309	0.01775

Table S9. Tabulated data for R^2, Reduced Chi-Sqr, τ and τ_{error} for PAA under different intensities of UV and Green irradiation.

**PAA intensity dependence**								
	**365nm**				455nm			
**Intensity**	**Adj. R²**	**Reduced Chi-Sqr**	**τ**	$\boldsymbol{\tau_{error}}$	Adj. R²	Reduced Chi-Sqr	τ	τ_{error}
mW/cm²								
0.25	0.99427	3.48E-04	9.41E-01	2.56E-03	0.99915	7.52E-07	2.35353	0.00119
0.5	0.98892	5.33E-04	1.62E+00	4.00E-03	0.99843	1.16E-06	1.88589	0.00128
1	0.9893	3.88E-04	1.13E+00	2.65E-03	0.99791	7.55E-07	0.88629	8.63E-04
2	0.98627	4.96E-04	6.04E-01	2.01E-03	0.99517	1.52E-06	0.37043	5.64E-04
4	0.984	5.54E-04	3.04E-01	1.48E-03	0.99094	1.79E-06	0.20971	4.42E-04
8	0.98145	5.97E-04	1.55E-01	1.09E-03	0.97042	2.88E-06	0.09791	3.73E-04

Table S10. Tabulated data for the rate constants for different wavelengths for polymer and bulk.

	I=1mWcm^{-2}			
λ	polymer		bulk	
	k_{TC}	k_{CT}	k_{TC}	k_{CT}
nm	cm^{-2}(mW·s)$^{-1}$			
365	0.0171	0.00163	0.03486	0.00137
455	0.00308	0.01778	0.00858	0.01287
490	8.53E-04	0.00639	0.00106	0.00463
530	1.20E-04	0.00138	1.13E-04	0.00102

Table S11. Tabulated data for R^2, Reduced Chi-Sqr, τ and τ_{error} for PAA under different wavelengths of irradiation.

PAA wavelength dependence				
Wavelength	Adj. R^2	Reduced Chi-Sqr	τ	τ_{error}
nm				
365	0.98701	1.25E-04	9.99E-01	1.12E-03
455	0.99573	6.00E-07	8.08E-01	7.17E-04
490	0.9877	3.52E-06	2.42E+00	2.65E-03
530	0.99831	5.50E-07	1.06E+01	1.92E-03

.

Section 2. Degree of ionization estimation

Titrations were performed with a commercial Titrando instrument (Metrohm) by measuring the pH as a function of the added volume of 0.1 M NaCl stock solution. The polymer concentration was adjusted to 1 wt %, and the starting pH was set to pH = 13 for PAA. The degree of ionization α was calculated via:

$$\alpha = \frac{V_{\mathrm{PH}} - V_{\mathrm{EP,2}}}{V_{\mathrm{EP,1}} - V_{\mathrm{EP,2}}}, \qquad \text{(S7)}$$

with added volume of stock solution at the measured pH V_{pH}, the volume at the first equivalence point $V_{\mathrm{EP,1}}$ (neutralization of excess acid or base), and the volume at the second equivalence point $V_{\mathrm{EP,2}}$ (neutralization of polymer).

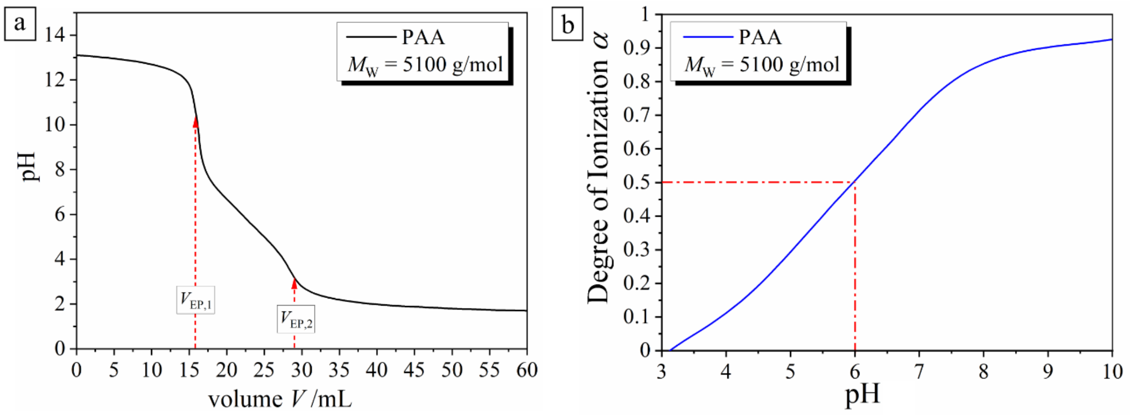

Figure S11. (a) Solution pH (aqueous PAA, c_{PAA} = 1 wt%) measured as a function of added volume of an aqueous HCl solution (c = 0.1 M). Arrows indicate the first and second equivalence point. Calculated degree of ionization from **Equation S7**. Note, that a reasonable degree of ionization of PAA cannot exceed values below 0 and over 1. .
